# Thirty-Day and One-Year All-Cause Mortality of ST-Segment Elevation Myocardial Infarction in Johannesburg, South Africa: Insights from the STEMI HOC-1 Prospective Study

**DOI:** 10.3390/jcdd12080282

**Published:** 2025-07-24

**Authors:** Marheb Badianyama, Arthur Mutyaba, Nqoba Tsabedze

**Affiliations:** Division of Cardiology, Department of Internal Medicine, School of Clinical Medicine, Faculty of Health Sciences, University of the Witwatersrand, Johannesburg 2193, South Africa

**Keywords:** ST-segment elevation myocardial infarction, acute coronary syndrome, thrombolysis, percutaneous coronary intervention, mortality, serum uric acid, South Africa, sub-Saharan Africa

## Abstract

Despite the increased mortality due to ST-segment elevation myocardial infarction (STEMI) in South Africa (SA), SA lacks comprehensive data on STEMI clinical outcomes. This study aimed to determine the 30-day and one-year all-cause mortality rates of STEMI patients presenting to our hospital. This was a one-year prospective single-centre study of STEMI patients presenting to the Charlotte Maxeke Johannesburg Hospital in SA between December 2021 and August 2023. We compared the baseline clinical characteristics, reperfusion strategies, and in-hospital, 30-day, and one-year clinical outcomes of survivors and non-survivors. This cohort included 378 STEMI participants. The in-hospital, 30-day, and one-year all-cause mortality rates were 6.6% (n = 25), 10.1% (n = 38), and 17.2% (n = 65), respectively. The pharmacoinvasive strategy was the most used reperfusion therapy (n = 150, 39.7%). On adjusted multivariate Cox regression analysis, a Killip class >2 was the strongest independent predictor of 30-day [HR 5.61, 95% CI 2.83–11.12; *p* < 0.001] and one-year all-cause mortality [HR 1.72, 95% CI 1.26–2.34; *p* = 0.001]. Although mortality has increased, our mortality rates were comparable to outcomes from high-income countries but significantly lower than reports from other low- or middle-income countries. Importantly, there were no significant differences in 30-day and one-year survival outcomes between the different reperfusion strategies.

## 1. Introduction

Ischaemic heart disease (IHD) is the leading cause of death globally [1]. ST-segment elevation myocardial infarction (STEMI) is the most lethal sequela of IHD, with at least half of deaths occurring before hospitalisation [2]. Provided there are no contraindications, the treatment of choice for a STEMI is timely coronary revascularisation with either thrombolysis, which offers maximum benefit within one hour from symptom onset, or primary percutaneous coronary intervention (PCI) performed 90 min from first medical contact [3]. In the absence of primary PCI and contraindications, thrombolysis can be provided within 12 h of symptom onset.

South Africa (SA) faces several barriers to achieving these treatment target times.

Therefore, an empirical alternative in our setting when primary PCI cannot be achieved within 90 min of first medical contact is the pharmaco-invasive strategy, which involves thrombolysis at a non-PCI centre followed by transfer to a PCI-administering centre for diagnostic coronary angiography (DCA) and/or rescue PCI within 24 h of thrombolysis. The pharmaco-invasive strategy offers similar all-cause mortality benefits to primary PCI and is superior to administering thrombolysis alone [4,5]. As such, in SA, revascularisation with either thrombolysis or a pharmaco-invasive strategy is a common practice [6,7].

Contrary to high-income countries (HIC), where death due to STEMI is declining, in sub-Saharan Africa (sSA), death due to cardiovascular diseases continues to increase and has superseded death due to communicable diseases, including the human immunodeficiency virus (HIV) infection [8,9]. The Second Euro Heart Survey has reported a 30-day mortality rate of 8.5% for STEMI [10]. In resource-limited African countries where access to PCI-capable facilities is limited, mortality rates are significantly higher, ranging between 22% and 28% [11,12]. However, the paucity of data, inequalities in access to high-quality healthcare, and heterogeneity of study results in LMIC make comparison of previously published data challenging.

The increase in STEMI-related deaths in SA is a public concern. Therefore, efforts must be made to identify factors contributing to an increased risk of STEMI-related deaths as well as attempts made to mitigate this risk. To our knowledge, there is no sizeable prospective data in South Africa on the one-year all-cause mortality outcomes of STEMI patients. In addition, despite the increased rate of death due to STEMI, South African data on the clinical predictors of 30-day and 1-year mortality in STEMI patients is scant.

Until recently, there has been increasing evidence that a raised serum uric acid (SUA) is both an independent risk factor for cardiovascular disease and an independent predictor of all-cause mortality in acute coronary syndromes (ACS) [13]. Uric acid is the final byproduct of purine metabolism synthesized by the enzyme xanthine oxidase in the human body. Increased xanthine oxidase (XO) activity and elevated SUA levels act as sources of reactive oxygen species (ROS), leading to increased oxidative stress, inflammation, and endothelial dysfunction in the cardiovascular system [14]. Previous data indicate an increase in SUA and XO activity during myocardial ischaemia and STEMI [15]. Furthermore, in the setting of STEMI treated with primary PCI, elevated SUA leads to larger reperfusion injury, coronary microvascular obstruction, and larger infarct size.

However, elevated SUA has also been associated with hypertension, diabetes mellitus, and obesity [14]. For this reason, evidence on whether SUA is a ‘true’ and independent risk factor of cardiovascular disease remains controversial, with previous studies showing conflicting results [13,14]. Previous research has reported that SUA in the hyperuricaemic and high-normal range of 0.30 mmol/L to 0.40 mmol/L (5.41 mg/dL to 7.21 mg/dL) independently predicts 30-day all-cause mortality of STEMI in both sexes [16]. Although there is yet to be a consensus on the cut-off value that defines an elevated SUA in IHD, multiple studies have defined this value to be equal to or greater than 0.38 mmol/L (≥6.8 mg/dL) in both sexes [17]. A systematic review and meta-analysis on the association of SUA and myocardial infarction, which included 2369 participants with STEMI, defined hyperuricemia as SUA levels equal to or greater than 0.39 mmol/L (7.0 mg/dL) for males and equal to or greater than 0.33 mmol/L (6.0 mg/dL) for females [18]. Despite the growing evidence of the role of SUA in acute myocardial infarction outcomes, to our knowledge, no study in SA has reported its significance as a baseline clinical prognostic factor of 30-day and 1-year all-cause mortality of STEMI.

This study presents real-world results from the ST-segment Elevation Myocardial Infarction Heart of Charlotte one-year (STEMI HOC-1) prospective study whose protocol has been previously published online [19]. The primary objective was to determine the all-cause mortality rate at 30 days and 1 year after the index acute STEMI presentation at the Charlotte Maxeke Johannesburg Academic Hospital (CMJAH) in Johannesburg, SA. We also compare the demographics, risk factor profile, biochemistry results, and echocardiographic and angiographic findings at baseline between survivors and non-survivors of acute STEMI. In addition, we report the time delays to ECG diagnosis and definitive treatment.

The secondary objective was to compare 30-day and 1-year mortality outcomes between thrombolysis, PCI, the pharmacoinvasive approach, and no reperfusion. We further describe the cardiovascular (CV) morbidity of STEMI in our setting by reporting the rates of planned or unplanned repeated revascularisation and the rates of the composite CV endpoint of angina, acute heart failure (HF), major bleeding episode, stroke or transient ischaemic attack (TIA), peripheral thromboembolism (i.e., pulmonary embolus, deep venous thrombosis), non-fatal arrhythmias (i.e., atrial fibrillation, ventricular tachycardia, and complete heart block), and CV-related re-hospitalisation at 30 days and one year after the index hospitalisation. Finally, we determine the baseline clinical predictors of 30-day and 1year all-cause mortality of acute STEMI, with particular interest in the clinical value of baseline SUA as a prognostic biomarker of 30-day and 1-year all-cause mortality in STEMI.

## 2. Materials and Methods

### 2.1. Study Design

This study was a single-centre, observational, prospective cohort of all consecutive patients presenting with an acute STEMI diagnosis. We followed patients over 360 days from the time of hospital discharge. The first day after hospitalisation was considered the first day of the follow-up.

### 2.2. Setting

The study was set in the Division of Cardiology at the Charlotte Maxeke Johannesburg Academic Hospital (CMJAH). The hospital is located in Johannesburg, SA, and is a 1088 beds state-owned tertiary referral healthcare facility with a catheterisation laboratory running daily from 08:00 to 16:00. As we are a referral hospital, we enrolled patients presenting directly to our emergency department (ED) and patients referred from peripheral primary and secondary-level healthcare facilities.

### 2.3. Study Definitions

We used the fourth universal definition of myocardial infarction for our case definition. An acute STEMI was defined as myocardial injury as indicated by a rise and/or fall of serum cardiac troponin levels with at least one value above the 99th percentile of the upper reference limit, in the presence of at least one of the following:

Ischaemic symptoms

New ischaemic electrocardiographic changes, i.e., persistent ST-segment elevation of at least 1 mm measured from the J-point in at least two contiguous leads, or new pathological Q-waves, or a new-onset left bundle branch block.

An acute STEMI diagnosis was confirmed with a 12-lead ECG. All patients were managed by cardiologists, and diagnoses were independently confirmed by the study investigators.

Primary PCI was not applicable to our third-world clinical setting. In instances where PCI was performed as the sole intervention, it was performed outside the recommended timeframe of 90 min. Hence, in this study, the term PCI refers to ‘non-primary’ or ‘late’ PCI performed long after 90 min of first medical contact without prior thrombolysis. Furthermore, the term ‘pharmacoinvasive strategy’ refers to the combination of thrombolysis and subsequent angiography and PCI.

Impaired renal function refers to abnormal results in serum creatinine, serum urea, and estimated glomerular filtration rate (GFR) calculated by the MDRD (Modification of Diet in Renal Disease Glomerular Filtration Rate) formula as a result of either an acute kidney injury or chronic kidney disease, irrespective of the underlying aetiology.

Menopause or post-menopause refers to the lack of menses for at least 12 consecutive months in an adult female, in the absence of contraceptive use.

### 2.4. Study Participants

We included all consecutive patients aged ≥ 18 years with a confirmed acute STEMI diagnosis who signed written informed consent. Participants or their next of kin needed to be reachable telephonically. We excluded individuals presenting with non-ST elevation acute coronary syndromes (NSTE-ACS), i.e., non-ST elevation myocardial infarction (NSTEMI) and unstable angina (UA), and those unreachable telephonically. From 10 December 2021 to 31 August 2023, 412 adults presented to our facility with an acute STEMI diagnosis. However, 27 were excluded due to misdiagnosis, death before giving consent, refusing consent, or not having a cellphone number. We enrolled 385 participants who underwent reperfusion with either intravenous thrombolysis or PCI or received conservative management. However, seven were lost during the study, and only 378 participants completed the 1-year cohort.

### 2.5. Data Sources

The data were collected prospectively during the index course of hospitalisation until discharge and at 30 and 360 days after the index discharge from the hospital. We sourced baseline clinical data from the hospital’s electronic patient medical record database. We retrieved all baseline blood laboratory results via the National Health Laboratory Service (NHLS) at the CMJAH.

The 30-day and 1-year clinical outcomes data were sourced through structured telephonic interviews using a standardised questionnaire to retrieve data on mortality, persistent angina, medication compliance, and cardiac-related re-admissions. The principal investigator (PI) enrolling the study participants conducted all telephonic interviews to minimise recall bias. If re-admitted to CMJAH, the researchers accessed the relevant hospital records to determine whether the study’s outcomes of interest had occurred. The modified data collection tools and structured interview questionnaires were developed from previous ACS registries and have been previously published with the study protocol [19].

### 2.6. Study Sample Size Calculations

A minimum sample size of 355 participants was required to provide the study with at least 80% power for statistical significance. We were able to enroll 385 consecutive participants, but only 378 records completed the one-year follow-up and form the basis of this report.

### 2.7. Statistical Analyses

We used StataCorp. LLC IC Version 16.1, College Station, TX, USA, for all statistical analysis. We expressed categorical data as numbers and percentages. We presented normally distributed continuous data as means with standard deviations (±SD). We reported continuously skewed data as medians with interquartile ranges (IQR). We compared survivors and non-survivors using Student’s unpaired *t*-test and one-way analysis of variance (ANOVA) with Fisher’s test for continuous variables and the chi-squared test for categorical variables. We presented the baseline characteristics in frequency tables. We used univariate and multivariate Cox proportional hazards regression analyses to determine the baseline clinical predictors of 30-day and 1-year all-cause mortality. Variables with a *p* < 0.10 on univariate analyses were included in the multivariate models. We expressed all variables with hazard ratios (HR) and 95% confidence intervals. For all tests, a *p*-value < 0.05 was considered statistically significant. We stored all research data in the Research Electronic Data Capture (REDCap) database hosted by the University of the Witwatersrand.

### 2.8. Ethical Approval and Consent to Participate

This study followed the Declaration of Helsinki. All study participants provided signed written informed consent at the time of admission before enrolling in the study and data extraction. The University of Witwatersrand Human Research Ethics Committee approved the study (certificate number M210427).

## 3. Results

**Figure 1:**
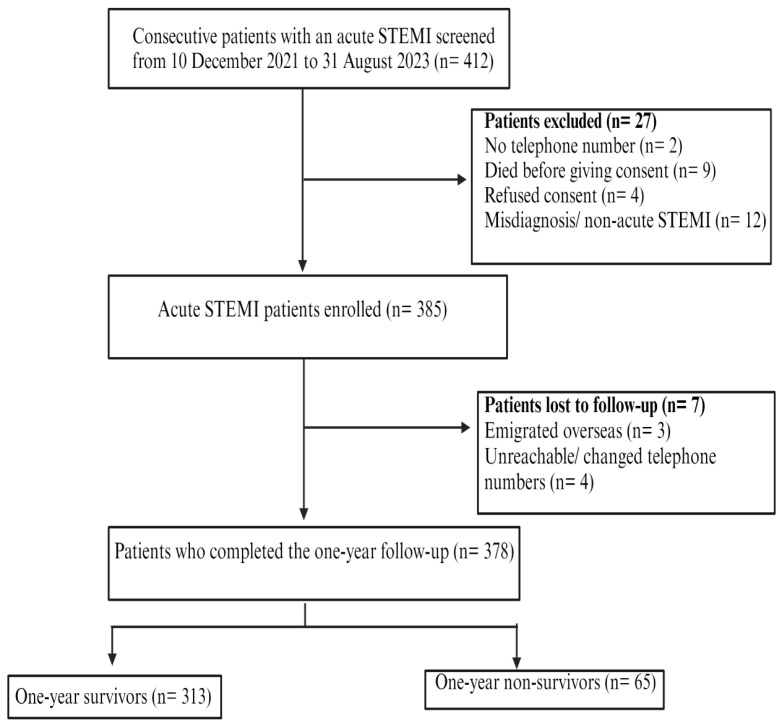
Study flow chart of acute STEMI patients presenting to the Charlotte Maxeke Johannesburg Academic Hospital (CMJAH) from December 2021 to August 2023.

The study recruitment occurred from 10 December 2021 to 31 August 2023 at the CMJAH. A total of 412 individuals presenting with an acute STEMI diagnosis were screened. Twenty-seven (27) adults were excluded due to lack of a cellphone number, death before consent, declined consent, misdiagnosis, or non-acute STEMI. We enrolled 385 eligible participants with a confirmed acute STEMI diagnosis. These participants were followed from admission to one year after the initial discharge date from the hospital. Seven patients were lost due to emigrating overseas (n = 3) and changing cellphone numbers (n = 4). Thus, only 378 patients completed the 1-year follow-up and were included in the study analysis of this report. Of the 378 patients followed, 313 (82.8%) survived, while 65 (17.2%) died during the cohort. Figure 1 summarises the study’s recruitment of participants.

### 3.1. Demographics and Risk Factor Profile

Of the 378 participants, 163 (43.1%) were Caucasian, 119 (31.5%) were Black, 82 (21.7%) were Indian, and 14 (3.7%) were of mixed race. The cohort was 77.5% male-dominant (n = 293), and the mean age was 56.2 ± 12.3 years. Patients who died at 30 days (63.4 ± 9.4 years) and one year (62.3 ± 11.4) were nearly ten years older than their surviving counterparts (55.4 ± 12.3 years and 54.9 ± 12.1 years, respectively; *p* < 0.001). Table 1 summarises the study participants’ demographics and risk factor profile stratified by mortality at 30 days and one year of the follow-up.

The five most common risk factors of atherosclerotic cardiovascular disease (ASCVD) in our study were hypertension (61.1%), active cigarette smoking (55.3%), dyslipidaemia (54.5%), diabetes mellitus (35.7%), and obesity (25.7%). However, one-year non-survivors had a significantly lower body mass index (BMI) at presentation (26.3 kg/m^2^, IQR 24.6–27.8) than one-year survivors (27.2 kg/m^2^, IQR 25.2–30.4; *p* < 0.042).

Of the total 85 women in the study, more than half (n = 46; 54.1%) were menopausal or post-menopausal. Notably, more than a quarter of the patients who died within 30 days of the study were menopausal or post-menopausal (26.3%) compared to the proportion of menopausal and post-menopausal women among the patients alive at 30 days (10.3%; *p* = 0.005).

Of the total cohort, thirty-three (8.7%) participants were HIV positive, and five of these (1.3%) died at one year. HIV-positive patients had baseline ASCVD risk factors similar to HIV-negative patients but were significantly younger (51.2 ± 9.7 years) than the remaining cohort (56.7 ± 12.4 years; *p* = 0.014). A history of a chronic kidney disease (CKD) diagnosis was a significantly common comorbidity among non-survivors (10.5%) compared to survivors (3.5%, *p* = 0.042) at 30 days and one year, respectively (9.2% vs. 10, 3.2%; *p* = 0.028).

### 3.2. Clinical Presentation, Time to ECG Diagnosis, and Diagnostic Clinical Investigations

Half of our cohort (n = 189) complained of persistent crushing chest pain at the time of presentation to our facility (Table 2). Nearly a quarter (23.1%) of one-year non-survivors had a history of Canadian Cardiovascular Society (CCS) chronic angina class three and four at presentation, compared to one-year survivors (10.2%; *p* = 0.004). The median time from symptom onset to ECG diagnosis was five hours (IQR 2.6–15.8). Inferior STEMI was the most common location of MI (38.4%, n = 145).

The median heart rate at admission was 85 beats per minute (bpm) (IQR 73–100), and one-year non-survivors had significantly higher heart rates at admission than one-year survivors (93 bpm, IQR 76–107 vs. 84 bpm, IQR 72–98, respectively; *p* = 0.029). Similarly, while the median systolic blood pressure at admission was 121.5 (IQR 106–137) mmHg for the entire cohort, non-survivors presented with significantly lower systolic blood pressures at baseline than survivors both at 30 days (113.5 mmHg, IQR 95–135 vs. 122 mmHg, IQR 107–138.5, respectively; *p* = 0.036) and one year (110 mmHg, IQR 98–131 vs. 123 mmHg, IQR 109–140, respectively; *p* = 0.001).

In biochemistry, the median peak high-sensitivity cardiac troponin T (hs-cTnT) levels on admission for non-survivors were nearly double (5411 ng/L, IQR 1818–8453 for 30-day non-survivors and 4942 ng/L, IQR 1818–8558 for one-year non-survivors, respectively) compared to survivors (2801 ng/L, IQR 1350.5–5645; *p* = 0.004 for 30 days survivors and 2729 ng/L, IQR 1336–5578; <0.001 for one-year survivors). Furthermore, one-year non-survivors had two times higher C-reactive protein (CRP) levels (28 mg/L, IQR 10–85) on admission compared to one-year survivors (14 mg/L, IQR 9–40; *p* = 0.006). Likewise, patients who died at one year presented with significantly higher levels of serum uric acid (SUA) (0.48 mmol/L, IQR 0.38–0.63) compared to those who survived (0.37 mmol/L, IQR 0.28–0.46; *p* < 0.001).

Furthermore, one-year non-survivors had significantly lower glomerular filtration rate (GFR) at baseline (64 mL/min/1.73 m^2^, IQR 44–87) compared to one-year survivors (89 mL/min/1.73 m^2^, IQR 69.4–112.2; *p* < 0.001). Similarly, the baseline potassium was significantly higher in one-year non-survivors (4.4 mmol/L, IQR 3.9–4.8) than in one-year survivors of the cohort (4.1 mmol/L, IQR 3.6–4.4; *p* < 0.001). Moreover, the baseline haemoglobin concentration was significantly lower in one-year non-survivors (14 g/dL, IQR 12.3–15.3) compared to their one-year surviving counterparts (14.8 g/dL, IQR 13.4–15.7).

As shown in Table 2, cardiogenic shock, sudden cardiac arrest, and Killip class > 2 at presentation were significantly associated with 30-day and one-year all-cause mortality (*p* < 0.001). Intermediate to high Global Regional Acute Coronary Events (GRACE) risk scores were more prevalent among non-survivors than survivors at 30 days (94.7% vs. 45.3%, *p* < 0.001) and one year (87.7% vs. 42.5%, *p* < 0.001), respectively. Furthermore, the median GRACE 2.0 risk of in-hospital mortality at baseline was significantly higher in 30-day non-survivors (9.4%, IQR 3.5–23.1) than in survivors (1.8%, IQR 1.1–3.7; *p* < 0.001) and in one-year non-survivors than in survivors (6.9%, IQR 3.2–22 vs. 1.6%, IQR 1.0–3.3; *p* < 0.001). Of the one-year non-survivors, 76.9% (n = 50) had a Thrombolysis in Myocardial Infarction (TIMI) risk score > 4 compared to 30% (n = 94) of the one-year survivors (*p* < 0.001).

More than two-thirds of non-survivors (68.4%) had an LVEF ≤ 40% at baseline echocardiography compared to those who survived at 30 days (47.9%, *p* = 0.017) and one year (66.2% vs. 46.7%, *p* = 0.004), respectively. The presence of a left ventricular clot on baseline echocardiography was significantly more prevalent in those who died compared to survivors at 30 days (7.9% vs. 4.1%, *p* = 0.006) and one year (7.7% vs. 3.8%, *p* = 0.034), respectively.

### 3.3. Coronary Reperfusion, Angiographic Findings, and Time Delays to Reperfusion

**Figure 2:**
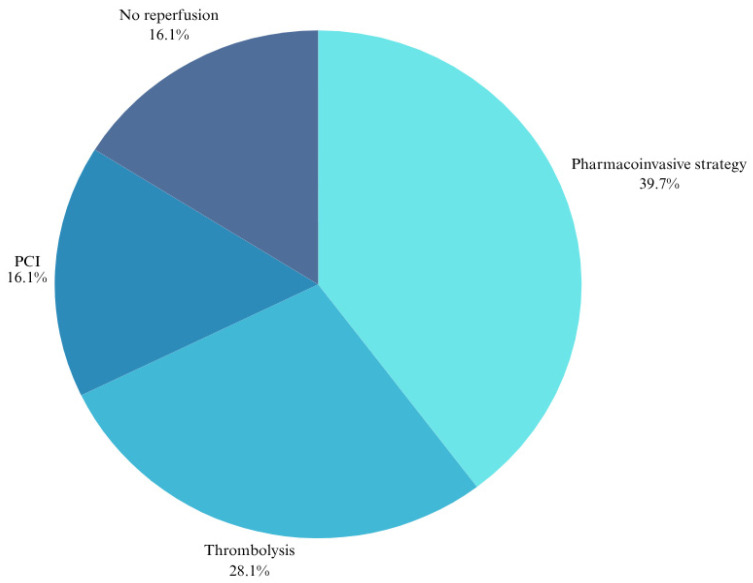
Coronary reperfusion strategies provided to patients presenting with an acute STEMI at the Charlotte Maxeke Johannesburg Academic Hospital (CMJAH) from December 2021 to August 2023. PCI, percutaneous coronary intervention.

As illustrated in Figure 2, of the 378 participants, 150 (39.7%) underwent pharmacoinvasive reperfusion, while 106 (28.1%) received thrombolysis exclusively. Sixty-one (16.1%) were reperfused by PCI only, and similarly, 61 patients (16.1%) received no coronary reperfusion but were managed with optimal medical therapy.

Alteplase was the most used thrombolytic agent (n = 172, 45.5%). The total number of DCA procedures performed in the cohort was 344 (91%) (Table 3). The right radial artery (RRA) was the most accessed primary vascular site for angiography (n = 289, 76.5%), and this was followed by the right femoral artery (RFA) (n = 39, 10.3%). In 12 subjects (3.2%), when vascular access through the RRA failed, operators converted to using the RFA as a secondary vascular access site.

On angiography, the left anterior descending (LAD) artery was the most common culprit coronary artery responsible for STEMI (n = 176, 46.6%). Occlusion of the left circumflex artery as the culprit lesion was the coronary vessel that was the least associated with one-year all-cause mortality (n = 0, *p* = 0.048). Approximately two-thirds of the cohort presented with less than three vascular occlusions on angiography (n = 249, 65.9%). Only 46 patients (12.2%) had triple vessel disease (TVD) or more, while 49 (13%) showed no evidence of coronary artery occlusion on angiography.

The total number of PCIs performed in the cohort was 211 (55.8%), either as part of pharmacoinvasive reperfusion or ‘non-primary’ PCI only. The most common PCI modality was percutaneous trans-luminal coronary angioplasty (PTCA) with subsequent drug-eluting stent (DES) implantation (n = 138, 36.5%). Forty subjects (10.6%) received a direct DES exclusively, while fourteen (3.7%) underwent PTCA only. Sixteen participants (4.2%) underwent aspiration thrombectomy, while six (1.6%) underwent PTCA with subsequent drug-eluting balloon (DEB) implantation. None of the participants who underwent direct DES died at 30 days (*p* = 0.025), and only one patient died at one year (*p* = 0.009). Direct DES was the PCI strategy associated with the least mortality at 30 days and one year (*p* = 0.025 and *p* = 0.009, respectively).

The median time from symptom onset to thrombolysis in this cohort was 6.2 h (IQR 3.8–10.8), and more than half of the cohort (n = 200, 52.9%) received thrombolysis within 12 h of symptom onset. The median time from symptom onset to PCI, irrespective of prior thrombolysis administration, was 64 h (IQR 32.2–110.4) without significant difference between survivors and non-survivors at 30 days (64.7 h, IQR 32.2–113.5 vs. 57.6 h, IQR 27.6–97.6; *p* = 0.559) and one year, respectively (66 h, IQR 36–111.8 vs. 55 h, IQR 25.4–106.1; *p* = 0.183). Only 12 patients (3.4%) underwent PCI within 12 h of symptom onset, none of which were primary PCI.

### 3.4. In-Hospital Clinical Outcomes and Length of Stay

The in-hospital all-cause mortality rate of the cohort was 6.6% (n = 25). As shown in Table 4, the composite CV endpoint occurred in 16.1% (n = 61) of the cohort during the index hospitalisation, and it was significantly higher in non-survivors compared to survivors at 30 days (60.5% vs. 11.2%, *p* < 0.001) and one year (41.5% vs. 10.9%, *p* < 0.001), respectively. In-hospital impaired renal function was nearly three times more commonly reported in patients who died than in those who survived at 30 days (39.5% vs. 12.7%, *p* < 0.001) and one year (32.3% vs. 11.8%, *p* < 0.001), respectively. The median duration of hospitalisation was 4 days (3–7) for the overall cohort without a significant difference between survivors and non-survivors at 30 days (4 days, IQR 3–7 vs. 4 days, IQR 2–8, *p* = 0.930) and one year, respectively (4 days, IQR 3–6 vs. 5 days, IQR 3–8, *p* = 0.108). All participants referred for coronary artery bypass graft (CABG) surgery (n = 8, 2.6%) survived one year (*p* = 0.039).

### 3.5. Mortality Outcomes of the Different Reperfusion Strategies

Coronary reperfusion by PCI only, which was, by definition, not primary PCI in terms of the guideline-recommended timeframe, was associated with no in-hospital mortality (n = 0, 0.0%) compared to thrombolysis (n = 12, 11.4%; *p* = 0.005), pharmacoinvasive reperfusion (n = 9; 6.0%; *p* = 0.047), and no reperfusion (n = 4, 6.7%; *p* = 0.037), which was statistically significant. However, there was no statistically significant difference in the 30-day and one-year mortality outcomes when comparing the individual reperfusion strategies, as shown in Table 5. Similarly, there were no statistically significant differences in the composite CV event rates between the different types of reperfusions.

### 3.6. Thirty-Day Clinical Outcomes

The 30-day all-cause mortality rate was 10.1% (n = 38). The incidence rate of 30-day mortality was 3.67 per 1000 person-days (95% CI 2.67–5.04) for the overall cohort of 378 subjects for a total of 10,361 person-days. The 30-day composite CV endpoint occurred in 89 subjects (23.5%) without significant differences in survivors and non-survivors (n = 83, 24.4% vs. n = 6, 15.8%, respectively; *p* = 0.235). Only 18 subjects (4.8%) underwent repeated revascularisation with staged PCI within 30 days of discharge from the hospital, of which 15 (83.3%) were planned and 3 (16.7%) were unplanned, with a significant difference between survivors and non-survivors (n = 15, 4.4% vs. n = 0, 0% for planned PCI and n = 1, 0.3% vs. n = 2, 5.3% for unplanned PCI; *p* = 0.001, respectively).

### 3.7. One-Year Clinical Outcomes

The one-year all-cause mortality rate was 17.2% (n = 65). The incidence rate of one-year mortality was 3.22 per 1000 person-days (95% CI 2.92–3.57) for the overall cohort of 378 subjects for a total of 117,219 person-days. The one-year composite CV endpoint occurred in 104 subjects (27.5%) with no significant difference between one-year survivors and non-survivors (n = 82, 26.2% vs. n = 22, 33.9%, respectively; *p* = 0.209).

### 3.8. Adherence to Guideline-Directed Medical Therapy (GDMT) at 30 Days and One-Year Follow-Up

The overall adherence rate to guideline-directed medical therapy (GDMT) was 92.8% (n = 323) at one year. The most prescribed GDMT at discharge was dual antiplatelet therapy (87.6%), followed by statins (85.7%), beta-blockers (81.5%), and angiotensin-converting enzyme inhibitors (ACE-I) (71.2%). Less than 2% of subjects were on aspirin or clopidogrel monotherapy. ACE-I had the highest non-adherence rate (4.3%) at 30 days, dropping from 71.2% at the time of discharge to 66.9% at 30 days. This non-adherence was chiefly due to the subjects reporting ACE-I adverse events such as angioedema and a dry cough. Consequently, an angiotensin receptor blocker (ARB) was prescribed as an alternative therapeutic agent. Statins had the highest non-adherence rate (5.2%) at one-year follow-up, dropping from 85.7% at discharge to 80.2% at one year.

### 3.9. Baseline Predictors of 30-Day Mortality

Although ‘non-primary’ PCI only (n = 59, 93.7%) had the highest rate of survival, on log-rank test, there was no statistically significant difference in 30-day survival rate between ‘non-primary’ PCI, pharmacoinvasive reperfusion (n = 137, 91.3%), thrombolysis only (n = 92, 87.6%), and those who received no reperfusion (n = 54, 90.0%) (*p* = 0.587).

The baseline predictors of 30-day all-cause mortality for patients with an acute STEMI are summarised in Table 6. On adjusted multivariate Cox regression analysis, the risk of 30-day mortality increased with increasing age [hazard ratio (HR) 1.05, 95% CI 1.02–1.08; *p* = 0.002]. A history of menopause or being a post-menopausal female more than doubled the risk of 30-day mortality [HR 2.31, 95% CI 1.10–4.86; *p* = 0.028] and independently predicted 30-day mortality after adjusting for race, hypertension, diabetes, dyslipidaemia, and a history of cigarette smoking on multivariate analysis.

A Killip class > 2 at clinical presentation independently increased the risk of death at 30 days by nearly six times compared to Killip class ≤ 2, even after adjusting for age and systolic blood pressure at baseline [HR 5.61, 95% CI 2.83–11.12; *p* < 0.001]. Similarly, a Thrombolysis in Myocardial Infarction (TIMI) risk score > 4 independently increased the risk of 30-day mortality by at least five-fold [HR 5.51, 95% CI 2.57–11.79; *p* < 0.001]. Notably, with every 1% increase in the Global Regional Acute Coronary Events (GRACE) 2.0 risk of in-hospital death, the risk of death at 30 days increased by 5% [HR 1.05, 95% CI 1.03–1.07; *p* < 0.001].

In addition, an LVEF ≤ 40% on admission doubled the risk of death at 30 days [HR 2.04, 95% CI 1.02–4.05; *p* = 0.043]. Tachycardia (i.e., heart rate ≥ 100 bpm) on presentation more than doubled the risk of 30-day mortality even after adjusting for age and baseline systolic blood pressure [HR 2.83, 95% CI 1.47–5.44; *p* = 0.002].

An increase in SUA at baseline was an independent predictor of 30-day mortality [HR 1.56, 95% CI 1.07–2.28; *p* = 0.022]. A raised CRP at baseline was associated with an increased risk of death at 30 days but not after adjusting for leukocyte counts at baseline on multivariate analysis [HR 1.01, 95% CI 1.00–1.01; *p* = 0.001].

In our cohort, hospitalization beyond 3 days could not independently predict the risk of 30-day mortality [HR 0.90, 95% CI 0.47–1.72; *p* = 0.743]. The times from the onset of symptoms to thrombolysis or PCI could not independently predict the risk of 30-day mortality [HR 0.98, 95% CI 0.94–1.03; *p* = 0.411, and HR 1.00, 95% CI 0.99–1.01; *p* = 0.719, respectively]. Most importantly, performing coronary angiography through the right radial artery as the primary vascular access site independently decreased the risk of 30-day mortality by 77%, even after adjusting for age, sex, risk factors of ASCVD, and time from ECG diagnosis to thrombolysis or PCI [HR 0.23, 95% CI 0.06–0.82; *p* = 0.024].

### 3.10. Baseline Predictors of One-Year Mortality

On the log-rank test, there was no statistically significant difference in one-year survival between those who only received thrombolysis (n = 81, 77.1%), those who underwent non-primary PCI (n = 54, 85.7%), those with a Pharmacoinvasive strategy (n = 128, 85.3%), and those who received no reperfusion (n = 50, 83.3%) in the cohort (*p* = 0.318).

An age of 80 years and above at clinical presentation independently predicted one-year all-cause mortality [HR 2.82, 95% CI 1.16–6.86; *p* = 0.023]. A Killip class > 2 at baseline nearly doubled the risk of one-year all-cause mortality [HR 1.72, 95% CI 1.26–2.34; *p* = 0.001]. A TIMI risk score > 4 at admission [HR 1.38, 95% CI 1.12–1.72; *p* = 0.003] and the baseline GRACE risk of one-year mortality calculated at baseline [HR 1.03, 95% CI 1.01–1.04; *p* < 0.001] were independent predictors of one-year all-cause mortality.

In our cohort, a baseline LVEF ≤ 40% was not an independent predictor of one-year all-cause mortality [HR 1.13, 95% CI 0.92–1.38; *p* = 0.252]. In addition, the times from the onset of symptoms to thrombolysis and PCI, respectively, could not predict one-year all-cause mortality [HR 1.00, 95% CI 0.99–1.00; *p* = 0.612 and HR 1.00, 95% CI 0.99–1.00; *p* = 0.857, respectively]. Table 7 shows the baseline predictors of one-year all-cause mortality for the cohort.

## 4. Discussion

### 4.1. Mortality Outcomes

In this prospective observational study of 378 subjects presenting with an acute STEMI at a large PCI-capable state-owned tertiary hospital in Johannesburg, South Africa, the in-hospital, 30-day, and one-year all-cause mortality rates were 6.6%, 10.1%, and 17.2%, respectively. The in-hospital, 30-day, and one-year mortality rates for acute STEMI have nearly tripled compared to the rates reported by the South African Acute Coronary Events Survey of Current Management Strategies (ACCESS) registry over two decades ago (2.6%, 2.4%, and 6.7%, respectively) [20]. This supports evidence that death due to STEMI in our region is increasing at a staggering rate, notwithstanding the increased clinical expertise available, access to PCI-capable facilities, and the relatively high rate of adherence to secondary prevention therapies.

Factors contributing to the increased mortality rates over the last two decades in our region include the increased burden of obesity, metabolic and cardiovascular-kidney-metabolic syndromes, and a sedentary lifestyle, as well as an increased life expectancy of people living with HIV following the advent of antiretroviral therapy, which together have led to an increased prevalence of risk factors of CV disease. Another contributing factor could be the sub-optimal chronic management of these CV risk factors despite relatively high adherence to GDMT, such as in our cohort, as well as the already implemented legislation on salt restrictions in consumable goods, sin taxation on alcohol and tobacco products, and the use of combination pills to treat chronic hypertension.

Compared to recent local South African data, although our in-hospital mortality rate was higher than reports from a prospective cohort including 284 acute STEMI patients in Tygerberg Hospital, situated in the Western Cape province of South Africa (4.9%), the 30-day mortality rates for acute STEMI patients in the two regions were similar (6.6% in our cohort vs. 6.7% in Tygerberg Hospital) [21]. Notably, the study in Tygerberg Hospital was conducted between July 2020 and March 2021, during the peak of the SARS-CoV-2 pandemic, when SA was in national lockdown. Therefore, those results may be an under-representation of STEMI-related mortality, as previous studies have reported a significant decline in the number of ACS admissions during that time period [22].

However, the in-hospital and 30-day mortality rates for acute STEMI in our study were significantly lower than data from other African countries such as Ethiopia, where the in-hospital and 30-day mortality rates were reported to be 26.1% and 31.5%, respectively [11]. In Fanta and colleagues’ Ethiopian study, none of the patients received thrombolysis despite being eligible, and only 9% underwent late PCI. This discrepancy between protocolized STEMI management and what is actually happening in LMIC underscores the dire state of ACS management across LMIC, where access to medical resources continues to be an unmet need.

Similarly, in a Tanzanian study by Hertz and colleagues, which enrolled 152 patients with an acute MI, the one-year all-cause mortality rate was 61.1% (n = 93) [23]. This staggeringly high mortality was attributed to the lack of thrombolysis at the presenting hospital, poor access to PCI-capable facilities, limited number of cardiologists, and lack of GDMT for secondary prevention. Furthermore, our in-hospital and one-year all-cause mortality rates (6.6% and 17.1%, respectively) were lower than reports from a large single-centre prospective Indian study including 2379 first-time acute STEMI patients, which reported in-hospital and one-year all-cause mortality rates of 11.1% and 22.6%, respectively [24].

### 4.2. Demographics

The patients in our study were young adults (56.2 ± 12.3 years) and predominantly males (77.5%) of Caucasian race (43.1%). However, the prevalence of STEMI among Black Africans has exponentially increased (31.5%) compared to data from the South African ACCESS registry reported more than a decade ago (5.9%) [20]. The increased burden of STEMI, particularly among Black African patients, supports data from previous studies reporting an epidemiological transition in sSA with the burden of non-communicable diseases (NCD) superseding that of communicable diseases [8].

The lower prevalence of STEMI among women compared to men in our study (22.5% vs. 77.5%, respectively) was consistent with reports from HICs and other LMICs such as India [24]. However, nearly a third of the 38 subjects who died within 30 days were menopausal or post-menopausal females compared to the proportion of menopausal and post-menopausal women among the patients who survived, which was statistically significant (26.3% and 10.6%; *p* = 0.005). Our findings are in keeping with previous studies, which have shown that CV disease is the leading cause of death in women, surpassing death due to breast and gynaecological cancers combined.

Notably, the risk of death due to IHD in women increases after menopause, and as such, this subgroup typically develops coronary artery disease a few years later than men [25]. Several gender-specific cardiometabolic factors secondary to menopause contribute to the increased risk of early mortality. These include a decline in estradiol and a rise in follicle-stimulating hormone leading to carotid vascular endothelial remodeling. Other factors include early-onset menopause and menopause caused by bilateral salpingo-oophorectomy with no hormonal replacement therapy compared to natural menopause. In addition, late peri-menopause and early post-menopause stages are associated with high levels of total cholesterol, high-density lipoprotein cholesterol (HDL-C), low-density lipoprotein cholesterol (LDL-C), triglycerides, and lipoprotein-A, which further increase CV disease risk. Furthermore, the presence of vasomotor symptoms during the various stages of menopause has been linked to insulin resistance, systemic hypertension, dyslipidaemia, and fat redistribution independently of estradiol and the traditional risk factors of ASCVD.

Furthermore, a history of menopause or post-menopause could independently predict the risk of 30-day mortality even after adjusting for other risk factors of ASCVD such as race, hypertension, diabetes, dyslipidaemia, and cigarette smoking [HR 2.31, 95% CI 1.10–4.86; *p* = 0.028]. Although being a menopausal or post-menopausal female increased the risk of one-year mortality, it could not independently predict the risk of one-year mortality in our study [HR 1.12, 95% CI 0.81–1.53; *p* = 0.489]. However, this lack of prediction could have been due to the relatively small number of women in the cohort (n = 85, 22.5%).

### 4.3. Risk Factor Profile and Comorbidities

Our cohort had a high prevalence of established risk factors of ASCVD. Our patients’ risk factor profile was different from other LMICs such as India, where diabetes (39.2%) and alcohol use (35.2%) are the two most prevalent risk factors of ASCVD, followed by active cigarette smoking (35.1%) and hypertension (34%), respectively [24]. Over the last decade, the prevalence of hypertension among STEMI patients in our region has increased by 20% compared to data from the South African ACCESS registry (61.1% now vs. 41.1% then) [20]. These results were consistent with the INTERHEART Africa study, which reported that a history of hypertension was the most vital modifiable risk factor associated with first-time acute MI in Black Africans [26]. The rate of active cigarette smoking (55.3%) was similar to data from the South African ACCESS registry (53.6%) [20]. However, it was alarmingly higher than reports from other sSA countries such as Ethiopia (14.4%) and Ivory Coast (29.3%) [11,27]. Therefore, aggressive interventions at a population level that focus on hypertension prevention and cigarette smoking cessation are crucial to mitigate the burden of ASCVD in South Africa and sSA.

HIV infection has been shown in previous studies to be associated with chronic inflammation leading to unstable atherosclerotic plaque morphology, dyslipidaemia independent of antiretroviral therapy (ART), as well as more severe coronary artery occlusion [28]. In this study, 33 STEMI patients (8.7%) had an HIV infection, all of whom were on combined ART. Previous South African studies have reported an HIV-positive rate of 6% among STEMI patients [21]. Despite the high prevalence of HIV in SA, the relatively low rates of HIV infection in these STEMI cohorts may be explained by the lack of routine HIV testing among individuals with CAD, potentially leading to under-reporting and underestimation of its prevalence in CAD. However, although HIV increases the risk of ASCVD, previous studies have reported that traditional CV risk factors have a more significant impact on mortality than HIV itself, especially in the presence of ART [29]. In our study, HIV was not associated with an increased risk of all-cause mortality.

### 4.4. Serum Cardiac Biomarkers of STEMI

Considerably, individuals who died at 30 days and one-year follow-ups had significantly higher levels of SUA at admission compared to their surviving counterparts. In this study, a raised SUA at admission was an independent predictor of 30-day mortality even after adjusting for traditional risk factors of ASCVD such as age, sex, hypertension, dyslipidaemia, diabetes, and cigarette smoking [HR 1.56, 95% CI 1.07–2.28; *p* = 0.022]. Therefore, our study’s results suggest that elevated SUA levels in acute STEMI may be both an independent CV risk factor and a poor prognostic marker of 30-day all-cause mortality.

Our results are supported by Mandurino-Mirizzi and colleagues’ large prospective study, which included 2369 STEMI patients and showed that elevated baseline SUA levels equal to or greater than 0.38 mmol/L (6.8 mg/dL) were an independent predictor of 30-day and one-year all-cause mortality after multivariate Cox regression analyses (HR 1.2, 95% CI 1.01–1.32; *p* = 0.042 and HR 1.18, 95% CI 1.05–1.32; *p* = 0.005, respectively) [17].

However, previous studies have debated whether SUA is a ‘true’ risk factor for CAD or a byproduct of this disease. Notably, in our cohort, subjects who died at 30 days and one year had a significantly higher prevalence of established chronic kidney disease (CKD) at admission compared to survivors (10.5% non-survivors vs. 3.5% survivors, *p* = 0.042 at 30 days and 9.2% non-survivors vs. 3.2% survivors, *p* = 0.028 at one year, respectively). While CKD has been shown to contribute to higher SUA concentrations, in this study, SUA was still found to be a strong independent predictor of 30-day mortality even after adjusting for traditional risk factors of ASCVD and CKD on multivariate analysis [HR 1.51, 95% CI 1.05–2.19; *p* = 0.028] [30]. Hence, our results show that SUA could be a ‘true’ risk factor for ASCVD.

However, in this study, we could not determine a standardised cut-off value that defines ‘high-normal’ or ‘high’ SUA levels predictive of 30-day and one-year mortality in acute STEMI. To our knowledge, there is yet to be a large randomised controlled trial determining a cut-off value for high SUA that is predictive of mortality in STEMI. Moreover, in our study, while a raised baseline SUA increased the risk of one-year mortality, it could not independently predict this risk [HR 1.20, 95% CI 0.96–1.50; *p* = 0.118]. An explanation for baseline SUAs lack of prediction of one-year mortality risk could have been our relatively small sample size.

### 4.5. Time Delays and Coronary Reperfusion Strategies Outcomes

Our rate of thrombolysis for the overall cohort (n = 255, 67.46%) was higher than in other LMICs such as India (45.8%), for example. However, there were significant time delays to thrombolysis. Previous South African data reported that factors contributing to delayed time to thrombolysis include patients being uneducated about the symptoms of an acute MI and the importance of early thrombolysis, underutilization and limited access to emergency medical services (EMS), and missed ECG diagnoses by physicians and other medical personnel [31,32]. This highlights the urgent need for widespread education campaigns on prompt recognition of an acute MI among the general public and healthcare professionals, as well as the establishment of structured referral networks between all levels of healthcare, which would improve the rates of coronary reperfusion and mitigate the risk of mortality in STEMI patients.

Furthermore, while 59.3% (n = 224) received thrombolysis at pre-PCI centres, less than 1% (n = 2) received it within one hour from the onset of symptoms of an acute MI. Our median time from symptom onset to thrombolysis (6.2 h, IQR 3.8–10.8) is similar to a recent study conducted at the Chris Hani Baragwanath Academic Hospital in SA, which enrolled 100 STEMI patients and reported a median time to thrombolysis of six hours (IQR 4.3–12.8) from the onset of symptoms [7]. Our delayed time to thrombolysis further corresponds with Meel and colleagues’ study on acute STEMI patients presenting to a tertiary hospital in Pretoria, SA, which found that only 3% were thrombolysed within one hour of hospital arrival [33]. Although our study did not investigate the reasons for these time delays, previous SA data have reported pre-hospital delays related to patient factors to be the main reasons for delays to reperfusion [7].

Moreover, the times from symptom onset to thrombolysis or PCI could not independently predict the risk of 30-day and one-year mortality. Reasons for this lack of prediction include our relatively small study sample size compared to HIC clinical trials, but it could also be that factors other than hospital time delays to coronary reperfusion play more significant roles in determining mortality outcomes. As shown in a previous study by Stassen and colleagues, although 53.8% and 71.5% of South Africans live within 60 and 120 min, respectively, of a PCI facility, primary PCI is still not administered within the recommended time frame of 90 min [34].

The median time from symptom onset to PCI, irrespective of prior thrombolysis administration, was 64 h (IQR 32.2–110.4), and only 12 patients (3.4%) underwent PCI within 12 h of symptom onset, none of which were primary PCI. Our significantly delayed median time from the onset of symptoms to PCI of 64 h (approximately 3 days) may have been attributed to patient and health system factors such as, for example, the lengthy referral pathway from primary to secondary then tertiary care. Geographical factors may have also played a role in delaying the time to timely PCI, as most of our patients are referred from remote health centres to our PCI-capable facility.

To reduce these delays and ultimately reduce mortality, several interventions need to be implemented, primarily at a national level. Some of these interventions include nationwide free patient educational programmes on the symptoms of an acute myocardial infarction, timely dispatching of ambulances equipped with an ECG machine for all patients complaining of chest pain, training of all EMS personnel to perform an ECG and recognize an acute STEMI on an ECG performed prior to arriving at a healthcare facility, increasing our country’s cardiologist-to-patients ratio by increasing the number of cardiology fellows trained at academic hospitals, and ultimately, the allocation of more funds toward healthcare infrastructure that includes a catheterization laboratory.

Our hospital’s rate of diagnostic coronary angiogram (DCA) procedures (91%) was comparable to data from HIC [35]. A reason for ‘non-primary’ PCI in this study to be associated with significant in-hospital mortality benefits compared to other strategies, despite PCI being performed beyond the recommended 90 min of first medical contact, may be attributed to cardiologists opting to perform this intervention in subjects with ongoing angina but who were haemodynamically stable, which could have been a confounding factor. However, in this study, there was no statistically significant difference in 30-day (*p* = 0.587) and one-year (*p* = 0.318) survival favoring a specific reperfusion strategy. The lack of survival benefits of ‘non-primary’ or ‘late’ PCI over the alternative reperfusion strategies in this study was consistent with previous studies evaluating late reperfusion, such as the Occluded Artery Trial (OAT), which randomised 2166 patients with stable myocardial infarctions to PCI versus no PCI group 3 to 28 days after the myocardial infarction and reported no difference in survival between the groups [36].

### 4.6. Baseline Clinical Predictors of 30-Day and One-Year All-Cause Mortality

In our study, the strongest baseline independent predictors of 30-day mortality were Killip class > 2, TIMI risk score > 4, in-hospital impaired renal function, tachycardia, menopausal or post-menopausal females, raised admission SUA, and an LVEF ≤ 40% at clinical presentation. Impaired renal function on admission was a strong independent baseline predictor of 30-day mortality, notwithstanding adjusting for a history of CKD on multivariate Cox regression analysis [HR 3.59, 95% CI 1.81–7.12; *p* < 0.001]. Our findings were in keeping with Ekou and colleagues’ Ivorian prospective study, which reported that impaired renal function was an independent predictor of 30-day mortality in patients with an acute MI (RR 3.44, 95% CI 1.63–7.26; *p* < 0.001] [37].

On the other hand, the strongest baseline-independent predictors of one-year mortality on multivariate Cox regression analysis were an age of 80 years and above (HR 2.82, 95% CI 1.16–6.86; *p* < 0.023), a Killip class > 2 (HR 1.72, 95% CI 1.26–2.34; *p* = 0.001), and a TIMI risk score > 4 (HR 1.38, 95% CI 1.12–1.72; *p* = 0.003).

Our study’s high adherence rate to GDMT one year after hospitalization (92.8%) was critical to reducing the rates of re-infarction and re-hospitalization in an already overburdened state-owned healthcare system. Patients who were not on optimal medical therapy at one-year follow-up were at higher risk of one-year mortality [HR 6.01, 95% CI 4.10–8.82; *p* < 0.001]. Hence, this further highlights the pertinent role that optimal pharmacotherapy plays in mitigating one-year mortality in acute STEMI.

### 4.7. Limitations

Our study had a low number of patients lost to follow-up (n = 7, 1.8%). Patients lost to follow-up were not included in the study’s statistical analyses. Nonetheless, this study had several limitations. Firstly, it was a single-centre study at a referral hospital and thus, prone to selection and referral bias. Secondly, although prospective, its observational nature did not allow us to control for potential unmeasured confounders. Thirdly, the follow-ups were performed telephonically and were thus prone to patient recall bias and under-reporting of adverse CV events of interest. However, to minimise recall bias, all telephonic interviews were conducted by the primary investigator, MB. Fourthly, in this study, when PCI was performed as the sole reperfusion strategy of choice, it was administered beyond the guideline-recommended timeframes. Finally, the time durations from the onset of symptoms to STEMI diagnosis and therapeutic interventions were drawn from times on ECG tracings and patient bed letters, respectively. Although we provided close estimates of these time delays, they may be longer than reported in this contemporary study. Nonetheless, our STEMI sample was sizeable compared to previous South African and other sub-Saharan African studies.

## 5. Conclusions

In this prospective study, the in-hospital, 30-day, and one-year all-cause mortality rates of acute STEMI were 6.6%, 10.1%, and 17.2%, respectively, which were similar to HIC but lower than other LMICs. In our region, acute STEMI affects young adults and most likely involves a single coronary vessel. Importantly, the prevalence of STEMI among Black Africans is rapidly increasing, and so is the prevalence of cardiovascular risk factors. The pharmacoinvasive strategy is the most common intervention in our setting, where primary PCI remains limited. Strong efforts must be made to provide primary PCI within the recommended timeframes to offer long-term mortality benefits. System delays are representative of the quality of care, and it is recommended that these are objectively measured and reduced. Future research on STEMI should focus on African-based randomised controlled trials that include a sizable female population and that provide more accurate timeframes to interventions and report the true prevalence and incidence of HIV infection in individuals with an acute STEMI, more particularly in the presence of HIV viral load suppression with combined ART. While raised SUA was an independent predictor of 30-day all-cause mortality, large randomised controlled trials are required to determine a standardised SUA cut-off value for males and females that could potentially be used as a low-cost prognostic biomarker of all-cause mortality in acute STEMI.

## Figures and Tables

**Table 1:** Demographics and risk factor profile of study participants who survived compared to those who died within 30 days and one year of follow-up.

		30-Day Follow-Up			1-Year Follow-Up		
Variables	Total N = 378 (%)	Alive N = 340 (89.9)	Dead N = 38 (10.1)	*p*-Value	Alive N = 313 (82.8)	Dead N = 65 (17.2)	*p*-Value
Demographics							
Male sex	293 (77.5)	267 (78.5)	26 (68.4)	0.157	246 (78.6)	47 (72.3)	0.269
Age, years ± SD	56.2 ± 12.3	55.4 ± 12.3	63.4 ± 9.4	<0.001 *	54.9 ± 12.1	62.3 ± 11.4	<0.001 *
Self-identified race							
Caucasian	163 (43.1)	139 (40.9)	24 (63.2)	0.071	130 (41.5)	33 (50.8)	0.581
Black	119 (31.5)	112 (32.9)	7 (18.4)		102 (32.6)	17 (26.2)	
Indian	82 (21.7)	76 (22.3)	6 (15.8)		69 (22.0)	13 (20.0)	
Mixed	14 (3.7)	13 (3.8)	1 (2.63)		12 (3.1)	12 (3.8)	
BMI, kg/m^2^ (IQR)	27 (25.1–30.1)	27 (25.1–30.2)	26.5 (24.2–30.1)	0.354	27.2 (25.2–30.4)	26.3 (24.6–27.8)	0.042 *
Abdominal circumference, cm (IQR)	88 (79–105)	88 (79–105)	86 (79–108)	0.862	88.5 (79.5–105)	86 (78–97.5)	0.207
Risk factors							
Hypertension	231 (61.1)	205 (60.3)	26 (68.4)	0.330	186 (59.4)	45 (69.2)	0.140
Diabetes mellitus	135 (35.7)	120 (35.3))	15 (39.5)	0.610	111 (35.5)	24 (36.9)	0.823
Active cigarette smoking	209 (55.3)	189 (55.6)	20 (52.6)	0.728	175 (55.9)	34 (52.3)	0.595
Cigarette smoking pack years (IQR)	20 (13–30)	20 (12.5–30)	32.5 (17.5–41)	0.016 *	20 (12.5–30)	25 (15–40)	0.155
Dyslipidaemia	206 (54.5)	187 (55.0)	19 (50.0)	0.557	177 (56.6)	29 (44.6)	0.079
Obesity (BMI ≥ 30.0)	97 (25.7)	90 (26.5)	7 (18.4)	0.281	86 (27.5)	11 (16.9)	0.076
CKD	16 (4.2)	12 (3.5)	4 (10.5)	0.042 *	10 (3.2)	6 (9.2)	0.028 *
PVD	10 (2.7)	7 (2.1)	3 (7.9)	0.034 *	7 (2.2)	3 (4.6)	0.277
Family history of MI	119 (31.5)	110 (32.3)	9 (23.7)	0.275	104 (33.2)	15 (23.1)	0.109
Menopause and post-menopause (if female sex)	46 (12.2)	36 (10.6)	10 (26.3)	0.005 *	34 (10.9)	12 (18.5)	0.088
Previous MI	56 (14.8)	48 (14.1)	8 (21.0)	0.254	43 (13.7)	13 (20.0)	0.196
Previous stroke/TIA	11 (2.9)	10 (2.9)	1 (2.6)	0.914	7 (2.2)	4 (6.2)	0.087
HIV infection	33 (8.7)	31 (9.1)	2 (5.3)	0.425	28 (8.9)	5 (7.7)	0.745
Other comorbidities (e.g., COPD, gout, malignancy)	77 (20.4)	67 (19.7)	10 (26.3)	0.337	57 (18.2)	20 (30.8)	0.022 *
Prior PCI	28 (7.4)	26 (7.6)	2 (5.3)	0.595	23 (7.3)	5 (7.7)	0.923
Prior CABG surgery	8 (2.1)	6 (1.8)	2 (5.3)	0.155	6 (1.9)	2 (3.1)	0.554

BMI, Body Mass Index; CABG, coronary artery bypass graft; CAD, coronary artery disease; CKD, chronic kidney disease; COPD, chronic obstructive pulmonary disease; HIV, human immunodeficiency virus; IQR, interquartile range; MI, myocardial infarction; PCI, percutaneous coronary intervention; PVD, peripheral vascular disease; TIA, transient ischaemic attack; * *p*-value < 0.05, statistically significant; ≥, greater than or equal to.

**Table 2:** Clinical presentation, time to ECG diagnosis, and diagnostic evaluations of study participants who survived compared to those who died within 30 days and one year of follow-up.

		30-Days Follow-Up			1-Year Follow-Up		
Variable	Total N = 378 (%)	Alive N = 340 (89.9)	Dead N = 38 (10.1)	*p*-Value	Alive N = 313 (82.8)	Dead N = 65 (17.2)	*p*-Value
Clinical presentation							
Crushing chest pain	189 (50.0)	164 (48.2)	25 (65.8)	0.040 *	154 (49.2)	35 (53.8)	0.496
Chronic angina CCS III-IV	47 (12.4)	40 (11.8)	7 (18.4)	0.238	32 (10.2)	15 (23.1)	0.004 *
Heart rate, bpm (IQR)	85 (73–100)	85 (73–98)	93 (70–111)	0.119	84 (72–98)	93 (76–107)	0.029 *
Systolic BP, mmHg (IQR)	121.5 (106–137)	122 (107–138.5)	113.5 (95–135)	0.036 *	123 (109–140)	110 (98–131)	0.001 *
Cardiogenic shock	29 (7.7)	17 (5.0)	12 (31.6)	<0.001 *	13 (4.2)	16 (24.6)	<0.001 *
Sudden cardiac arrest	16 (4.2)	10 (3.0)	6 (15.8)	<0.001 *	8 (2.6)	8 (12.3)	<0.001 *
Killip class > II	50 (13.2)	32 (9.4)	18 (47.4)	<0.001 *	23 (7.4)	27 (41.5)	<0.001 *
GRACE risk score intermediate to high risk	190 (50.3)	154 (45.3)	36 (94.7)	<0.001 *	133 (42.5)	57 (87.7)	<0.001 *
GRACE 2.0 risk of in-hospital death, (%) (IQR)	2.1 (1.1–4.2)	1.8 (1.1–3.7)	9.4 (3.5–23.1)	<0.001 *	1.6 (1.0–3.3)	6.9 (3.2–22)	<0.001 *
TIMI score > 4	144 (38.1)	115 (33.8)	29 (76.3)	<0.001 *	94 (30.0)	50 (76.9)	<0.001 *
Time from symptom onset to ECG diagnosis, hours (IQR)	5.0 (2.6–15.8)	5.2 (2.8–16.7)	3.6 (1.9–8.4)	0.068	5.0 (2.6–15.8)	4.8 (2.4–20.2)	0.761
Key laboratory findings							
Peak hs-cTnT, ng/L (IQR)	2935 (1436–5861)	2801 (1350.5–5645)	5411 (1818–8453)	0.004 *	2729 (1336–5578)	4942 (1818–8558)	<0.001 *
CRP, mg/L (IQR)	14.4 (9–53)	14 (9–44.5)	43 (9–136)	0.015 *	14 (9–40)	28 (10–85)	0.006 *
Serum uric acid, mmol/L (IQR)	0.38 (0.29–0.48)	0.38 (0.29–0.47)	0.48 (0.38–0.59)	<0.001 *	0.37 (0.28–0.46)	0.48 (0.38–0.63)	<0.001 *
Urea, mmol/L (IQR)	5.7 (4.5–7.6)	5.5 (4.4–7.3)	9.4 (6.2–14)	<0.001 *	5.5 (4.3–7)	7.8 (6–13.2)	<0.001 *
Creatinine, μmol/L (IQR)	89 (74–107)	87 (73–101.5)	112.5 (83–188)	<0.001 *	86 (73–101)	102 (82–141)	<0.001 *
GFR, mL/min/1.73 m^2^ (IQR)	84.9 (65–109)	87 (68.1–111)	54 (33–79)	<0.001 *	89 (69.4–112.2)	64 (44–87)	<0.001 *
Haemoglobin, g/dL (IQR)	14.7 (13.2–15.7)	14.8 (13.4–15.7)	13.6 (12.2–15)	0.016 *	14.8 (13.4–15.7)	14 (12.3–15.3)	0.019 *
Echocardiography							
LVEF ≤ 40%	189 (50.0)	163 (47.9)	26 (68.4)	0.017 *	146 (46.7)	43 (66.2)	0.004 *
LVRWMA	352 (93.1)	316 (92.9)	36 (94.7)	0.678	292 (93.3)	60 (92.3)	0.776
LV thrombus	17 (4.5)	14 (4.1)	3 (7.9)	0.006 *	12 (3.8)	5 (7.7)	0.034 *

CCS, Canadian Cardiovascular Society grading of angina pectoris; CRP, C-reactive protein; ECG, electrocardiogram; GFR, glomerular filtration rate calculated by the Modification of Diet in Renal Disease (MDRD) equation in mL/min/1.73 m^2^; GRACE, Global Registry of Acute Coronary Events risk score; hs-cTnT, high-sensitivity cardiac troponin T; IQR, interquartile range; LV, left ventricle; LVEF, left ventricular ejection fraction; LVRWMA, left ventricular regional wall motion abnormality; SUA, serum uric acid; TIMI, Thrombolysis In Myocardial Infarction risk score. * *p*-value < 0.05, statistically significant; >, greater than; <, less than.

**Table 3:** Coronary angiographic findings and time parameters in patients who survived compared to those who died within 30 days and one year of follow-up.

		30 Days Follow-Up			1-Year Follow-Up		
Variables	Total N = 378 (%)	Alive N = 340 (89.9)	Dead N = 38 (10.1)	*p*-Value	Alive N = 313 (82.8)	Dead N = 65 (17.2)	*p*-Value
Angiographic findings							
Total number of DCA procedures performed	344 (91.0)	318 (93.5)	26 (68.4)	<0.001 *	297 (94.9)	47 (72.3)	<0.001 *
Coronary angiography vascular access site							
RRA	289 (76.5)	269 (79.1)	20 (52.6)	<0.001 *	252 (80.5)	37 (56.9)	<0.001 *
LRA	1 (0.3)	1 (0.3)	0 (0.0)	0.738	1 (0.3)	0 (0.0)	0.828
RFA	39 (10.3)	34 (10.0)	5 (13.2)	0.544	32 (10.2)	7 (10.8)	0.895
Failed RRA converted to RFA	12 (3.2)	11 (3.2)	1 (2.6)	0.840	9 (2.9)	3 (4.6)	0.467
LFA	1 (0.3)	1 (0.3)	0 (0.0)	0.738	1 (0.3)	0 (0.0)	0.828
Failed RRA converted to LFA	1 (0.3)	1 (0.3)	0 (0.0)	0.738	1 (0.3)	0 (0.0)	0.828
Culprit occluded coronary artery							
LAD	176 (46.6)	162 (47.7)	14 (36.8)	0.205	149 (47.6)	27 (41.5)	0.372
RCA	104 (27.5)	92 (27.1)	12 (31.6)	0.554	87 (27.8)	17 (26.2)	0.787
Left circumflex artery	18 (4.8)	18 (5.3)	0 (0.0)	0.146	18 (5.8)	0 (0.0)	0.048 *
Left main stem artery	3 (0.8)	3 (0.9)	0 (0.0)	0.561	3 (1.0)	0 (0.0)	0.428
Number of coronary arteries with > 70% stenosis							
Less than three vessels occluded	249 (65.9)	230 (67.6)	19 (50.0)	0.128	217 (69.3)	32 (49.2)	0.454
TVD or more	46 (12.2)	40 (11.8)	6 (15.8)		37 (11.8)	9 (13.8)	
No coronary lesions	49 (13.0)	48 (14.1)	1 (2.6)		43 (13.7)	6 (9.2)	
PCI modality							
PTCA and DES	138 (36.5)	125 (36.8)	13 (34.2)	0.756	116 (37.1)	22 (33.9)	0.624
PTCA and DEB	6 (1.6)	5 (1.5)	1 (2.6)	0.587	3 (1.0)	3 (4.6)	0.032 *
PTCA only	14 (3.7)	10 (2.9)	4 (10.5)	0.019 *	10 (3.2)	4 (6.2)	0.250
Direct DES only	40 (10.6)	40 (11.8)	0 (0.0)	0.025 *	39 (12.5)	1 (1.5)	0.009 *
Aspiration thrombectomy	16 (4.2)	15 (4.4)	1 (2.6)	0.605	14 (4.5)	2 (3.1)	0.611
Time parameters, hours							
Time from symptom onset to thrombolysis, hours (IQR)	6.2 (3.8–10.8)	6.5 (3.8–11.3)	4.6 (3.4–7.9)	0.136	6.5 (3.8–12)	4.8 (3.4–7.9)	0.108
Time from symptom onset to PCI, hours (IQR)	64 (32.2–110.4)	64.7 (32.2–113.5)	57.6 (27.6–97.9)	0.559	66 (36–111.8)	55 (25.4–106.1)	0.183
Time from ECG diagnosis to PCI, hours (IQR)	50.0 (23.5–87.4)	51.4 (23.5–89.3)	34.6 (23.5–66)	0.299	53.3 (24.5–90)	29.0 (17.0–68.2)	0.041 *
Time from symptom onset to thrombolysis							
≤12 h	200 (52.9)	178 (52.4)	22 (57.9)	0.516	161 (51.4)	39 (60.0)	0.208
>12–24 h	34 (9.0)	32 (9.4)	2 (5.3)	0.397	31 (9.9)	3 (4.6)	0.175
>24–72 h	16 (4.2)	14 (4.1)	2 (5.3)	0.739	13 (4.2)	3 (4.6)	0.866
>72 h	5 (1.3)	5 (1.5)	0 (0.0)	0.452	5 (1.6)	0 (0.0)	0.387
Time from symptom onset to PCI							
≤12 h	13 (3.4)	13 (3.8)	0 (0.0)	0.220	10 (3.2)	3 (4.6)	0.567
>12–24 h	18 (4.8)	16 (4.7)	2 (5.3)	0.878	15 (4.8)	3(4.6)	0.951
>24–72 h	90 (23.8)	80 (23.5)	10 (26.3)	0.702	76 (24.3)	14 (21.5)	0.637
>72 h	91 (24.1)	85 (25.0)	6 (15.8)	0.208	80 (25.6)	11 (16.9)	0.138

DCA, diagnostic coronary angiogram; DEB, drug-eluting balloon; DES, drug-eluting stent; IQR, interquartile range; LAD, left anterior descending artery; LFA, left femoral artery; LRA, left radial artery; PCI, percutaneous coronary intervention; PTCA, percutaneous transluminal coronary angioplasty; RCA, right coronary artery; RFA, right femoral artery; RRA, right radial artery; TVD, triple vessel disease. * *p*-value < 0.05, statistically significant; ±, with or without; >, greater than; ≥, greater than or equal to; ≤, less than or equal to.

**Table 4:** In-hospital clinical outcomes and length of stay of patients who survived compared to patients who died in the cohort.

		30 Days Follow-Up			1-Year Follow-Up		
Variable	Total N = 378 (%)	Alive N = 340 (89.9%)	Dead N = 38 (10.1%)	*p*-Value	Alive N = 313 (82.8%)	Dead N = 65 (17.2%)	*p*-Value
Composite in-hospital CV endpoint ^a^	61 (16.1)	38 (11.2)	23 (60.5)	<0.001 *	34 (10.9)	27 (41.5)	<0.001 *
Impaired renal function	58 (15.3)	43 (12.7)	15 (39.5)	<0.001 *	37 (11.8)	21 (32.3)	<0.001 *
Repeated coronary angiography (i.e., staged PCI/relook)	29 (7.7)	28 (8.2)	1 (2.6)	0.218	24 (7.7)	5 (7.7)	0.995
Length of stay, days (IQR)	4 (3–7)	4 (3–7)	4 (2–8)	0.930	4 (3–6)	5 (3–8)	0.108
LOS ≤ 3 days	145 (38.4)	130 (38.2)	15 (39.5)	0.882	124 (39.6)	21 (32.3)	0.270
LOS > 3 days	233 (61.6)	210 (61.8)	23 (60.5)		189 (60.4)	44 (67.7)	
Referral for CABG surgery	8 (2.1)	8 (2.4)	0 (0.0)	0.597	8 (2.6)	0 (0.0)	0.039 *

^a^ Composite in-hospital cardiovascular endpoint includes angina, non-fatal arrhythmias, acute heart failure, major bleeding, stroke, transient ischaemic attack, and thromboembolic events; CABG, coronary artery bypass graft; CV, cardiovascular; IQR, interquartile range; LOS, length of stay; PCI, percutaneous coronary intervention; ≤, equal to or less than; >, more than; * *p*-value < 0.05, statistically significant.

**Table 5:** Outcomes of the different reperfusion strategies.

Reperfusion Type	Thrombolysis (A) n = 105 (%)	PCI (B) n = 63 (%)	PIS (C) n = 150 (%)	No Reperfusion (D) n = 60 (%)	Total n = 378	A vs. B *p*-Value	B vs. C *p*-Value	A vs. C *p*-Value	A vs. D *p*-Value	B vs. D *p*-Value	C vs. D *p*-Value
Total deaths	24 (22.9)	9 (14.3)	22 (14.7)	10 (16.7)	65 (17.2)	N/A	N/A	N/A	N/A	N/A	N/A
Of which in-hospital	12 (11.4)	0 (0.0)	9 (6.0)	4 (6.7)	25 (6.6)	0.005 *	0.047 *	0.121	0.320	0.037 *	0.856
Of which within 30 days of discharge	2 (1.9)	5 (7.9)	4 (2.7)	2 (3.6)	13 (3.4)	0.087	0.101	0.745	0.603	0.312	0.787
Of which after 30 days of discharge but within one year	10 (9.5)	4 (6.3)	9 (6.0)	4 (6.7)	27 (7.1)	0.861	0.427	0.502	0.818	0.713	0.749
^a^ Composite CV endpoint at 30 days	28 (26.7)	13 (20.6)	33 (22.0)	15 (25.0)	89 (23.5)	0.378	0.825	0.390	0.815	0.564	0.640
^a^ Composite CV endpoint at one year	33 (31.4)	15 (23.8)	40 (26.7)	16 (26.7)	104 (27.5)	0.290	0.664	0.408	0.520	0.715	1.000

^a^ Composite in-hospital cardiovascular endpoint includes angina, non-fatal arrhythmias, acute heart failure, major bleeding, stroke, transient ischaemic attack, and thromboembolic events. (A) Refers to thrombolysis; (B) refers to ‘non-primary’ percutaneous coronary intervention (PCI); (C) refers to pharmacoinvasive strategy (PIS); (D) refers to no reperfusion or optimal medical therapy; CV, cardiovascular; N/A, non-applicable; * *p*-value < 0.05, statistically significant.

**Table 6:** Clinical predictors of 30-day all-cause mortality of acute STEMI patients enrolled in the cohort study.

Clinical Predictors of 30-Day All-Cause Mortality				
	Univariate Analysis		Multivariate Analysis	
Variable	Unadjusted HR (95% CI)	*p*-Value	Adjusted HR (95% CI)	*p*-Value
Age, years	1.05 (1.02–1.08)	<0.001 *	1.05 (1.02–1.08)	0.002 *
Female sex	1.61 (0.81–3.19)	0.174	1.55 (0.77–3.10)	0.218
Race				
Caucasian	2.55 (1.10–5.92)	0.029 *	1.72 (0.72–4.10)	0.224
Indian	1.24 (0.42–3.70)	0.694	0.97 (0.32–2.93)	0.963
Mixed	1.21 (0.15–9.83)	0.859	0.70 (0.08–5.79)	0.740
SBP < 90 mmHg	3.56 (1.57–8.09)	0.002 *	3.44 (1.52–7.83)	0.003 *
Heart rate ≥ 100, bpm	2.42 (1.28–4.59)	0.007 *	2.83 (1.47–5.44)	0.002 *
Menopausal or post-menopausal female	2.65 (1.29–5.45)	0.008 *	2.31 (1.10–4.86)	0.028 *
Killip > II	6.49 (3.43–12.30)	<0.001 *	5.61 (2.83–11.12)	<0.001 *
TIMI risk score > 4	5.48 (2.60–11.59)	<0.001 *	5.51 (2.57–11.79)	<0.001 *
GRACE 2.0 in-hospital mortality risk (%)	1.06 (1.04–1.08)	<0.001 *	1.05 (1.03–1.07)	<0.001 *
GRACE one year mortality risk (%)	1.05 (1.04–1.07)	<0.001 *		
LVEF ≤ 40%	2.21 (1.12–4.38)	0.023 *	2.04 (1.02–4.05)	0.043 *
Peak hs-cTnT, ng/L	1.00 (0.99–1.00)	0.074	1.00 (0.99–1.00)	0.077
Baseline Hb, g/dL	0.87 (0.76–0.99)	0.036 *	0.88 (0.77–1.01)	0.070
CRP, mg/L	1.00 (1.00–1.01)	0.001 *	1.01 (1.00–1.01)	0.001 *
Serum uric acid, mmol/L	1.65 (1.19–2.31)	0.003 *	1.56 (1.07–2.28)	0.022 *
Creatinine, μmol/L	1.00 (1.00–1.01)	<0.001 *	1.00 (1.00–1.01)	<0.001 *
GFR, mL/min/1.73 m^2^	0.97 (0.96–0.98)	<0.001 *	0.98 (0.96–0.99)	0.046 *
LOS > 3 days	0.95 (0.50–1.82)	0.878	0.90 (0.47–1.72)	0.743
Time from symptom onset to thrombolysis, hours	0.99 (0.95–1.03)	0.475	0.98 (0.94–1.03)	0.411
Time from symptom onset to PCI, hours	1.00 (0.99–1.01)	0.476	1.00 (0.99–1.01)	0.719
RRA access site	0.33 (0.17–0.62)	0.001 *	0.23 (0.06–0.82)	0.024 *
Impaired renal function	3.83 (2.00–7.34)	<0.001 *	3.59 (1.81–7.12)	<0.001 *
Number of cigarette smoking pack years, years	1.02 (1.01–1.04)	0.010 *	1.01 (1.00–1.03)	0.188

CI, confidence interval; CRP, C-reactive protein; GFR, glomerular filtration rate calculated by the Modification of Diet in Renal Disease (MDRD) equation in mL/minute/1.73 m^2^; GRACE, Global Registry of Acute Coronary Events risk score; HF, heart failure; HR, hazard ratio; hs-cTnT, high-sensitivity cardiac troponin T; LOS, length of stay; LVEF, left ventricular ejection fraction; PCI, percutaneous coronary intervention; RRA, right radial artery; SBP, systolic blood pressure; TIMI, Thrombolysis In Myocardial Infarction risk score; * *p*-value < 0.05, statistically significant. >, more than; <, less than; ≥, more than or equal to; ≤, less than or equal to.

**Table 7:** Clinical predictors of one-year all-cause mortality of acute STEMI patients enrolled in the cohort study.

Clinical Predictors of One-Year All-Cause Mortality				
	Univariate Analysis		Multivariate Analysis	
Variable	Unadjusted HR (95% CI)	*p*-Value	Adjusted HR (95% CI)	*p*-Value
Age ≥ 80 years	2.88 (1.19–6.98)	0.019 *	2.82 (1.16–6.86)	0.023 *
Female sex	1.06 (0.83–1.35)	0.636	1.07 (0.84–1.36)	0.594
Race				
Caucasian	1.07 (0.85–1.36)	0.572	1.00 (0.78–1.28)	0.997
Indian	1.02 (0.77–1.35)	0.905	0.98 (0.74–1.30)	0.894
Mixed	1.00 (0.58–1.74)	0.993	0.91 (0.52–1.60)	0.738
Heart rate, bpm	1.00 (1.00–1.01)	0.416	1.00 (1.00–1.01)	0.321
Menopausal or post-menopausal females	1.13 (0.83–1.53)	0.448	1.12 (0.81–1.53)	0.489
Killip class > II	1.78 (1.32–2.40)	<0.001 *	1.72 (1.26–2.34)	0.001 *
TIMI score > 4	1.38 (1.12–1.70)	0.002 *	1.38 (1.12–1.72)	0.003 *
LVEF ≤ 40%	1.13 (0.92–1.38)	0.239	1.13 (0.92–1.38)	0.252
GRACE risk of in-hospital mortality	1.03 (1.02–1.04)	<0.001 *	1.02 (1.01–1.04)	<0.001 *
GRACE risk of one-year mortality, %	1.02 (1.01–1.03)	<0.001 *	1.03 (1.01–1.04)	<0.001 *
Baseline creatinine, mmol/L	1.00 (1.00–1.01)	<0.001 *	1.00 (1.00–1.01)	<0.001 *
GFR, mL/min/1.73 m^2^	1.00 (0.99–1.00)	0.023 *	1.00 (0.99–1.00)	0.024 *
CRP, mg/L	1.00 (0.99–1.00)	0.145	1.00 (0.99–1.00)	0.163
Serum uric acid, mmol/L	1.19 (0.96–1.47)	0.112	1.20 (0.96–1.50)	0.118
Peak hs-cTnT, ng/L	1.00 (0.99–1.00)	0.361	1.00 (0.99–1.00)	0.325
RRA access	0.81 (0.63–1.02)	0.074	0.87 (0.56–1.35)	0.532
Time from symptom onset to thrombolysis, hours	1.00 (0.99–1.00)	0.686	1.00 (0.99–1.00)	0.612
Time from symptom onset to PCI, hours	1.00 (0.99–1.00)	0.810	1.00 (0.99–1.00)	0.857
LOS > 3 days	1.05 (0.85–1.29)	0.668	1.03 (0.83–1.27)	0.785

CI, confidence interval; CRP, C-reactive protein; GFR, glomerular filtration rate calculated by the Modification of Diet in Renal Disease (MDRD) equation in mL/minute/1.73 m^2^; GRACE, Global Registry of Acute Coronary Events risk score; HF, heart failure; HR, hazard ratio; hs-cTnT, high-sensitivity cardiac troponin T; LOS, length of stay; LVEF, left ventricular ejection fraction; PCI, percutaneous coronary intervention; RRA, right radial artery; TIMI, Thrombolysis In Myocardial Infarction risk score; * *p*-value < 0.05, statistically significant. >, more than; ≥, more than or equal to; ≤, less than or equal to.

## Data Availability

The data used in this study is available from the corresponding author on reasonable grounds. The study protocol and its data collection tools have previously been published online [19].

## Abbreviations

ASCVD	atherosclerotic cardiovascular disease
CV	cardiovascular
DCA	diagnostic coronary angiography
ECG	electrocardiogram
IHD	ischaemic heart disease
HIC	high-income countries
LMIC	low-or middle-income countries
PCI	percutaneous coronary intervention
SA	South Africa
sSA	sub-Saharan Africa
STEMI	ST-elevation myocardial infarction
SUA	serum uric acid

## References

[1] World Health Organization (2020). The Top 10 Causes of Death: WHO. https://www.who.int/news-room/fact-sheets/detail/the-top-10-causes-of-death.

[2] Toshima T., Hirayama A., Watanabe T., Goto J., Kobayashi Y., Otaki Y., Wanezaki M., Nishiyama S., Kutsuzawa D., Kato S. (2021). Unmet needs for emergency care and prevention of prehospital death in acute myocardial infarction. J. Cardiol..

[3] Byrne R.A., Rossello X., Coughlan J.J., Barbato E., Berry C., Chieffo A., Claeys M.J., Dan G.-A., Dweck M.R., Galbraith M. (2023). 2023 ESC Guidelines for the management of acute coronary syndromes. Eur. Heart J..

[4] Fazel R., Joseph T.I., Sankardas M.A., Pinto D.S., Yeh R.W., Kumbhani D.J., Nallamothu B.K. (2020). Comparison of Reperfusion Strategies for ST-Segment–Elevation Myocardial Infarction: A Multivariate Network Meta-analysis. J. Am. Heart Assoc..

[5] Armstrong P.W., Gershlick A., Goldstein P., Wilcox R., Danays T., Bluhmki E., Van de Werf F. (2010). The Strategic Reperfusion Early After Myocardial Infarction (STREAM) study. Am. Heart J..

[6] Ndaba L., Mutyaba A., Mpanya D., Tsabedze N. (2023). In-Hospital Mortality Outcomes of ST-Segment Elevation Myocardial Infarction: A Cross-Sectional Study from a Tertiary Academic Hospital in Johannesburg, South Africa. J. Cardiovasc. Dev. Dis..

[7] Tickley I., Van Blydenstein S.A., Meel R. (2023). Time to thrombolysis and factors contributing to delays in patients presenting with ST-elevation myocardial infarction at Chris Hani Baragwanath Academic Hospital, Johannesburg, South Africa. SAMJ S. Afr. Med. J..

[8] Mayosi B.M., Flisher A.J., Lalloo U.G., Sitas F., Tollman S.M., Bradshaw D. (2009). The burden of non-communicable diseases in South Africa. Lancet.

[9] Sliwa K., Wilkinson D., Hansen C., Ntyintyane L., Tibazarwa K., Becker A., Stewart S. (2008). Spectrum of heart disease and risk factors in a black urban population in South Africa (the Heart of Soweto Study): A cohort study. Lancet.

[10] Mandelzweig L., Battler A., Boyko V., Bueno H., Danchin N., Filippatos G., Gitt A., Hasdai D., Hasin Y., Marrugat J. (2006). The second Euro Heart Survey on acute coronary syndromes: Characteristics, treatment, and outcome of patients with ACS in Europe and the Mediterranean Basin in 2004. Eur. Heart J..

[11] Fanta K., Daba F.B., Asefa E.T., Melaku T., Chelkeba L., Fekadu G., Gudina E.K. (2021). Management and 30-Day Mortality of Acute Coronary Syndrome in a Resource-Limited Setting: Insight From Ethiopia. A Prospective Cohort Study. Front. Cardiovasc. Med..

[12] Desta D.M., Nedi T., Hailu A., Atey T.M., Tsadik A.G., Asgedom S.W., Kasahun G.G., Ayalew E. (2020). Treatment outcome of acute coronary syndrome patients admitted to Ayder Comprehensive Specialized Hospital, Mekelle, Ethiopia; A retrospective cross-sectional study. PLoS ONE.

[13] Wang H., Yang J., Sao J., Zhang J., Pang X. (2018). The prediction of cardiac events in patients with acute ST segment elevation myocardial infarction: A meta–analysis of serum uric acid. Open Life Sci..

[14] Demiray A., Afsar B., Covic A., Kuwabara M., Ferro C.J., Lanaspa M.A., Johnson R.J., Kanbay M. (2022). The Role of Uric Acid in the Acute Myocardial Infarction: A Narrative Review. Angiology.

[15] Kroll K., Bukowski T.R., Schwartz L.M., Knoepfler D., Bassingthwaighte J.B. (1992). Capillary endothelial transport of uric acid in guinea pig heart. Am. J. Physiol.-Heart Circ. Physiol..

[16] Trkulja V., Car S. (2012). On-admission serum uric acid predicts outcomes after acute myocardial infarction: Systematic review and meta-analysis of prognostic studies. Croat. Med. J..

[17] Mandurino-Mirizzi A., Cornara S., Somaschini A., Demarchi A., Galazzi M., Puccio S., Montalto C., Crimi G., Ferlini M., Camporotondo R. (2021). Elevated serum uric acid is associated with a greater inflammatory response and with short- and long-term mortality in patients with ST-segment elevation myocardial infarction undergoing primary percutaneous coronary intervention. Nutr. Metab. Cardiovasc. Dis..

[18] Rong J., Fang C., Chen X., Hong C., Huang L. (2023). Association of serum uric acid with prognosis in patients with myocardial infarction: An update systematic review and meta-analysis. BMC Cardiovasc. Disord.

[19] Badianyama M., Mutyaba A., Nel S., Tsabedze N. (2023). ST-segment elevation myocardial infarction heart of Charlotte one-year (STEMI HOC-1) study: A prospective study protocol. BMC Cardiovasc. Disord..

[20] Schamroth C. (2012). Management of acute coronary syndrome in South Africa: Insights from the ACCESS (Acute Coronary Events—A Multinational Survey of Current Management Strategies) registry. Cardiovasc. J. Afr..

[21] Cilliers J.C.D., Joubert L., Beyers B., Ngarande E., Herbst P., Doubell A., Pecoraro A. (2023). The incidence and outcomes of high-risk acute coronary syndromes in Western Cape Province, South Africa: A prospective cohort study. S. Afr. Med. J..

[22] Joubert L.H., Herbst P.G., Doubell A.F., Pecoraro A.J.K. (2020). Clinical v. laboratory-based screening for COVID-19 in asymptomatic patients requiring acute cardiac care. S. Afr. Med. J..

[23] Hertz J.T., Sakita F.M., Kweka G.L., Limkakeng A.T., Galson S.W., Ye J.J., Tarimo T.G., Temu G., Thielman N.M., Bettger J.P. (2020). Acute myocardial infarction under-diagnosis and mortality in a Tanzanian emergency department: A prospective observational study. Am. Heart J..

[24] Paul G.J., Sankaran S., Saminathan K., Iliyas M., Sethupathy S., Saravanan S., Prabhu S.S., Kurian S., Srinivas S., Anurag P. (2023). Outcomes of ST Segment Elevation Myocardial Infarction without Standard Modifiable Cardiovascular Risk Factors—Newer Insights from a Prospective Registry in India. Glob. Heart.

[25] El Khoudary S.R., Aggarwal B., Beckie T.M., Hodis H.N., Johnson A.E., Langer R.D., Limacher M.C., Manson J.E., Stefanick M.L., Allison M.A. (2020). Menopause Transition and Cardiovascular Disease Risk: Implications for Timing of Early Prevention: A Scientific Statement From the American Heart Association. Circulation.

[26] Steyn K., Sliwa K., Hawken S., Commerford P., Onen C., Damasceno A., Ounpuu S., Yusuf S. (2005). Risk factors associated with myocardial infarction in Africa: The INTERHEART Africa study. Circulation.

[27] N’Guetta R., Yao H., Ekou A., N’Cho-Mottoh M.P., Angoran I., Tano M., Konin C., Coulibaly I., Anzouan-Kacou J.B., Seka R. (2016). Prevalence and characteristics of acute coronary syndromes in a sub-Saharan Africa population. Ann. Cardiol. D’angeiologie.

[28] Bahrami H., Budoff M., Haberlen S.A., Rezaeian P., Ketlogetswe K., Tracy R., Palella F., Witt M.D., McConnell M.V., Kingsley L. (2016). Inflammatory Markers Associated With Subclinical Coronary Artery Disease: The Multicenter AIDS Cohort Study. J. Am. Heart Assoc..

[29] Hyle E.P., Mayosi B.M., Middelkoop K., Mosepele M., Martey E.B., Walensky R.P., Bekker L.-G., Triant V.A. (2017). The association between HIV and atherosclerotic cardiovascular disease in sub-Saharan Africa: A systematic review. BMC Public Health.

[30] Johnson R.J., Sanchez Lozada L.G., Lanaspa M.A., Piani F., Borghi C. (2023). Uric Acid and Chronic Kidney Disease: Still More to Do. Kidney Int. Rep..

[31] Uys F., Beeton A.T., van der Walt S., Lamprecht M., Verryn M., Vallie Y., Stokes D., Millar R.S., Viljoen C.A. (2022). Profile and management of acute coronary syndromes at primary- and secondary-level healthcare facilities in Cape Town. Cardiovasc. J. Afr..

[32] Mabuza L.H., Mntla P.S. (2020). Generalist practitioners’ self-rating and competence in electrocardiogram interpretation in South Africa. Afr. J. Prim. Health Care Amp Fam. Med..

[33] Meel R., Gonçalves R. (2015). Time to fibrinolytics for acute myocardial infarction: Reasons for delays at Steve Biko Academic Hospital, Pretoria, South Africa. S. Afr. Med. Journal..

[34] Stassen W., Wallis L., Vincent-Lambert C., Castren M., Kurland L. (2018). The proportion of South Africans living within 60 and 120 minutes of a percutaneous coronary intervention facility. Cardiovasc. J. Afr..

[35] Belle L., Cayla G., Cottin Y., Coste P., Khalife K., Labèque J.N., Farah B., Perret T., Goldstein P., Gueugniaud P.Y. (2017). French Registry on Acute ST-elevation and non-ST-elevation Myocardial Infarction 2015 (FAST-MI 2015). Design and baseline data. Arch. Cardiovasc. Dis..

[36] Hochman J.S., Lamas G.A., Buller C.E., Dzavik V., Reynolds H.R., Abramsky S.J., Forman S., Ruzyllo W., Maggioni A.P., White H. (2006). Coronary Intervention for Persistent Occlusion after Myocardial Infarction. N. Engl. J. Med..

[37] Ekou A., Kipenge R., Yao H., Ehouman E., Touré C., Vy L., N’Guetta R. (2024). Thirty-day and one-year outcomes and predictors of mortality following acute myocardial infarction in Côte d’Ivoire: Data from the REACTIV survey. Arch. Cardiovasc. Dis..

